# Dr. Andrey’s Case

**Published:** 1886-03-20

**Authors:** A. Jacobi


DR. ANDREY'S CASE.—Since the article of Dr. Andrey, contained in the original department of this issue, was printed we have received from him the following letter by Dr. A. Jacobi, of New York, in response to a letter asking his opinion as to the diagnosis of the case, and give it place here, not being able to publish it in connection with the original account of the case, as would have been done if received in time :

110 S. 34TH STREET, NEW YORK, MARCH 7, 1886.

DR. J. A. ANDREY :

Dear Sir—As your patient was dead I was not so pressed for an answer as if the latter could have been of any use to the little child.

The child has died undoubtedly with pulmonary œdema ("suffocative catarrh"). But this is always a *secondary* condition. The contracted pupils, and the early unconsciousness, point also to cerebral œdema ("hyperæmia of the brain"). This is also a condition secondary to something else (primary). It is possible to attribute the pulmonary œdema to the paralysis brought on *by* the cerebral œdema ; but it is very much more probable that the two conditions were the co ordinate results of something *behind them.*

There are two things uppermost in my mind as possible causes—

1st. Attack of intermittens, which sometimes assumes this form in little children, even in districts not particularly cursed with very bad forms of malaria.

2d. Nephritis. It will often run its course with but few symptoms, or none that we observed. Dizziness, headaches, are sometimes noticed as symptoms by those who can give an account of themselves. The fatal attacks may be quite sudden, with or without convulsions.

You say nothing of the urine, of the temperature, of a post-mortem.

Very respectfully yours, A. JACOBI, M. D.

